# Mediastinal Hibernoma: A Rare Cause of Chronic Cough

**DOI:** 10.7759/cureus.6738

**Published:** 2020-01-22

**Authors:** Bernardo J Muñoz-Palacio, Shairine Figueroa, Gustavo Matute, Carolina Garcia-Mejía, Juan F Betancur

**Affiliations:** 1 Pulmonology, Clinica Medellin, Medellin, COL; 2 Internal Medicine, Clinica Las Américas, Medellin, COL; 3 Pathology, Clinica Medellin, Medellin, COL; 4 Family Medicine, Clinica Medellin, Medellin, COL; 5 Internal Medicine, Clinica Medellin Occidente, Medellin, COL

**Keywords:** hibernoma, mediastinal tumor, brown fat tissue

## Abstract

A hibernoma is an uncommon benign soft tissue tumor composed of brown adipose cells; the mediastinal location as presentation is scarce, with only six cases previously reported. The diagnosis of hibernoma is challenging and must be made based on the clinical, radiographic, and cytologic features. Here we present a 33-year-old woman without any relevant medical history presented for outpatient evaluation of a dry cough persisting for three months, and the X-rays revealed a dense well-defined mass with smooth borders in the left upper posterior mediastinum. Posterior mediastinal lesions represent a relatively small proportion of patient loads in thoracic surgery and account for a total of 25% of the cases, with neurogenic tumors among the most frequently seen in adults. Of these, the nerve sheath tumors (schwannoma, neurofibroma, paraspinal ganglioneuroma) are the most seen. Other differential diagnoses of paravertebral masses are the paraspinal abscess, metastases, hematoma, descending aortic aneurysm, among others. The patient underwent surgical resection via left posterolateral thoracotomy, without complications.

## Introduction

A hibernoma is an uncommon benign soft tissue tumor composed of brown adipose cells. Their brownish color is due to the presence of numerous mitochondria, with a high concentration of cytochrome pigments. Unlike white adipose tissue, which primarily serves as an energy store, brown adipose tissue serves primarily to create heat. Merkel first described this type of tumor in 1906 who called it a pseudolipoma and subsequently reported by Gery in 1914 as hibernoma due to its microscopic similarity to the brown fat of hibernating animals [1]. In humans, brown adipose tissue usually occurs in the fetus, being distributed in the neck, axillae, and subpleural regions, and gradually decrease. In adults, brown fat is confined to the more central parts of the body, with a vest-like distribution: along the esophagus, trachea, large vessels of the mediastinum, posterior neck, and interscapular regions [2]. However, despite this, mediastinal location is uncommon, with only six cases reported in the literature [2-7]. Notwithstanding their benign behavior, some variants of hibernoma can be confused histologically with liposarcoma, and this is why, the diagnosis must be made based on the clinical, radiographic, and cytologic features.

## Case presentation

A 33-year-old woman without any relevant medical history, only bilateral breast implants, presented for outpatient evaluation of a dry cough persisting for three months. The patient denied shortness of breath, chest pain, fever, chills, weight loss, or any other related symptoms. Gastroesophageal reflux, upper airways nasal syndrome, and tuberculosis were excluded. The patient does not smoke. She works as a nurse but denied unprotected contact with patients exhibiting respiratory symptoms.

Physical examination revealed normal vital signs. Cardiovascular examination revealed a normal cardiac rhythm without murmurs, rubs, or gallops. The pulmonary examination does not reveal any abnormal breathing pattern, without dyspnea or use of accessory muscles; there are no peripheral signs of respiratory dysfunction: cyanosis or clubbing, no abnormalities in the shape of thorax or asymmetry of chest expansion. Lungs were clear to auscultation, with vesicular breathing, without pathological breath sounds. Tactile fremitus was normal; no percussion dull note was noted. Complete blood count (CBC) and serum chemistry results were unremarkable; serial sputum acid-fast mycobacteria stain and cultures were negative.

**Figure 1:**
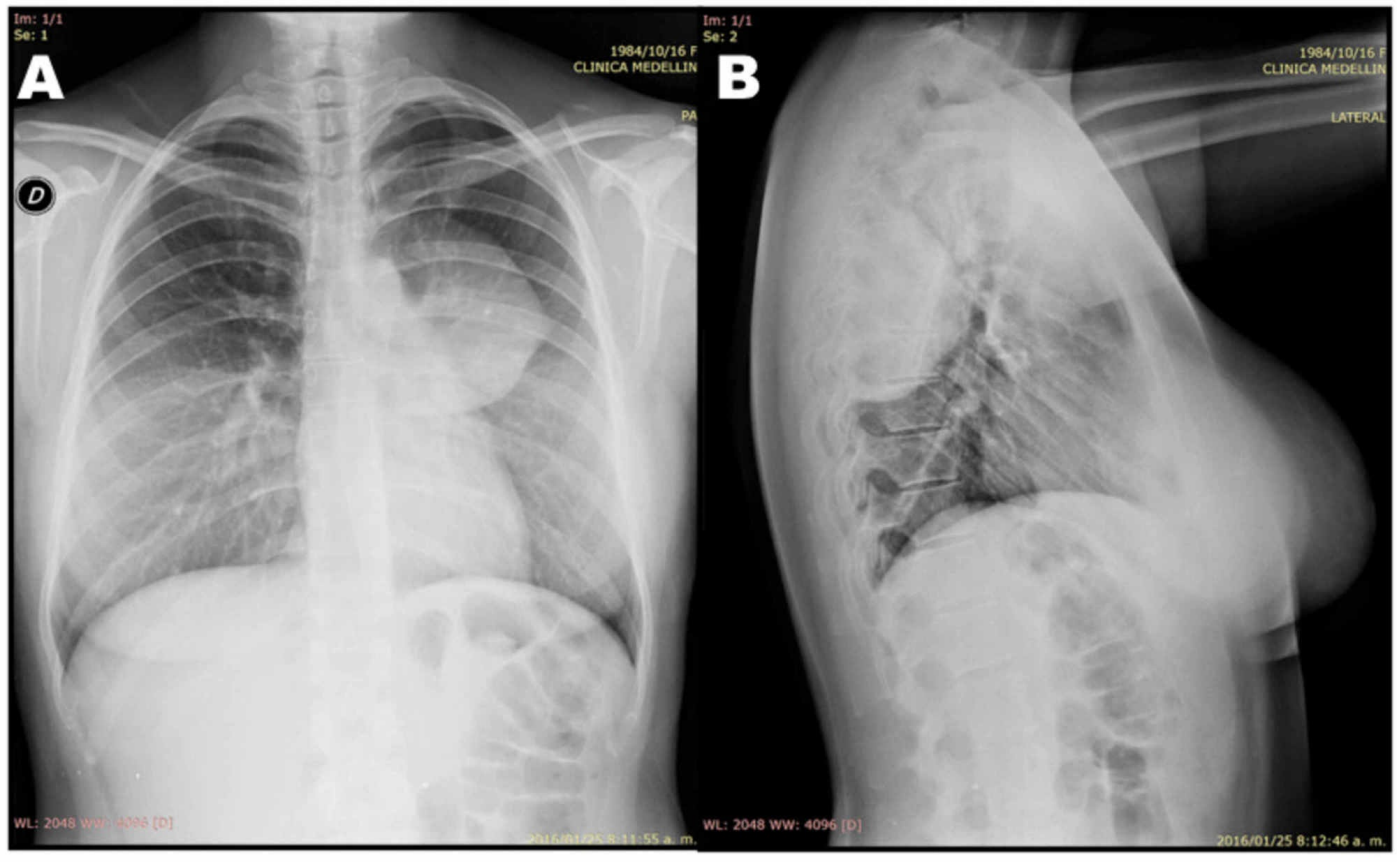
Posteroanterior and lateral CXR. Posteroanterior (A) and lateral (B) CXR showing a dense well-defined mass located on the left posterior mediastinum, with smooth borders, a minimal reaction in the adjacent lung tissue, acute angle borders, with no rib erosion of the overlying mass, or enlarged neural foramina. Breast implants are noted. CXR, chest X-ray

**Figure 2:**
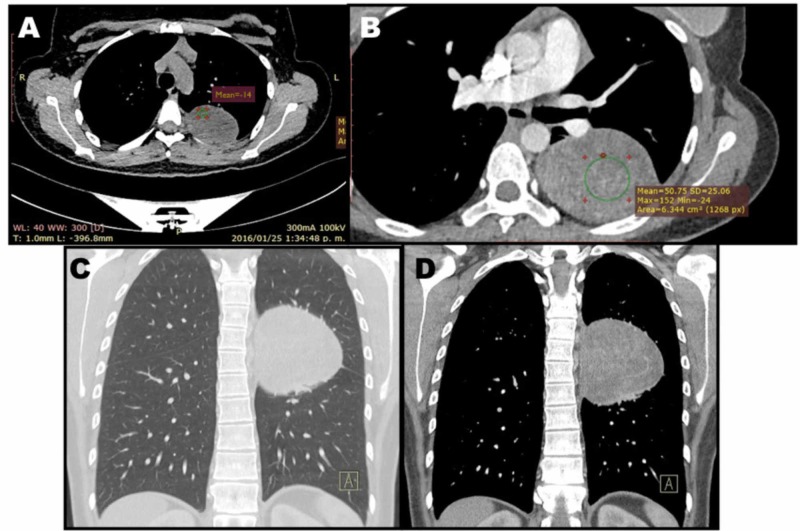
Chest CT scan. A. Axial pre-contrast CT scan of the chest showing a lesion attached to the parietal pleura, heterogeneous with areas of lower density (-14 HU) indicating the presence of macroscopic fat. Adjacent passive atelectasis was seen. B. Axial post-contrast CT scan showing heterogeneous enhancement going from an average of 16 to 55 HU indicating areas of hypervascularity. There is no widening of the neural foramina or origin in the medullary canal indicating the noncontiguity with the dorsal roots. No bone erosions indicating slow growth of benign appearance. No large vessels are irrigating the lesion. C. Coronal reconstruction of the CT scan showing the mass. D. Mediastinal window of the CT scan.

The posteroanterior chest X-ray (CXR) (Figure 1A) revealed a dense well-defined mass with smooth borders in the left upper mediastinum, cardiac contour, and hilar vessels which can be seen through the mass, placing it in the posterior mediastinum; the lateral X-ray (Figure 1B) confirms its location. The mass had a minimal reaction within the adjacent lung tissue, no rib erosion adjacent to the mass, and no expansion of the neural foramen. No calcification, volume loss, consolidation, or pleural effusion. For better mass characterization, a contrast-enhanced chest CT was performed. The CT showed a well-circumscribed mass of 8.1 cm x 7.8 cm x 6.3 cm (longitudinal-anteroposterior-transverse, respectively) located in the superior segment of the inferior lobe of the left lung (Figure 2). This mass was in intimate contact with the parietal pleura. Pre-contrast images showed a heterogeneous mass with lower density areas-14 Houndsfield units (HU) (Figure 2A). The mass had an average of 16 HU on pre-contrast images and went up to 55 HU post-contrast suggesting hypervascularity (Figure 2B). The mass made contact with the left inferior lobe pulmonary artery, and to a lesser degree, with the descending thoracic aorta, without compressing or invading them. There was no mediastinal lymphadenopathy, overt invasion into adjacent structures, or widening of the neural foramen. A radiologic diagnosis of neurogenic origin tumor was made.

Magnetic resonance imaging was not considered necessary due to sufficient data on CT images, and the cleavage margin was observed, and it did not change the surgical conduct.

Biopsy before surgery was not considered due to the risk of tumor implantation.

The patient underwent a left posterolateral thoracotomy through the fifth intercostal space. The tumor was encapsulated and adhered to the third intercostal space, over the emergence of the nerve root, and compromised the posterior segment of the upper left lobe and the superior segment of the inferior lobe. An upper lung decortication with posterior parietal pleurectomy over the mediastinum was made to leave a margin of resection of the tumor lesion with segmentary lobectomy of the posterior upper lobe and the superior segment of the inferior lobe due to the compromise of the visceral pleura. An open thoracic approach was made considering their strategic position in the mediastinum and the size of the tumor >5 cm. Although it is worth clarifying that the posterior mediastinum location is not a contraindication for a video-assisted thoracoscopy (VATS), it is a widely accepted treatment modality, and the size >5 cm is a relative contraindication as there are reports of lesion resections up to 18 cm in diameter.

**Figure 3:**
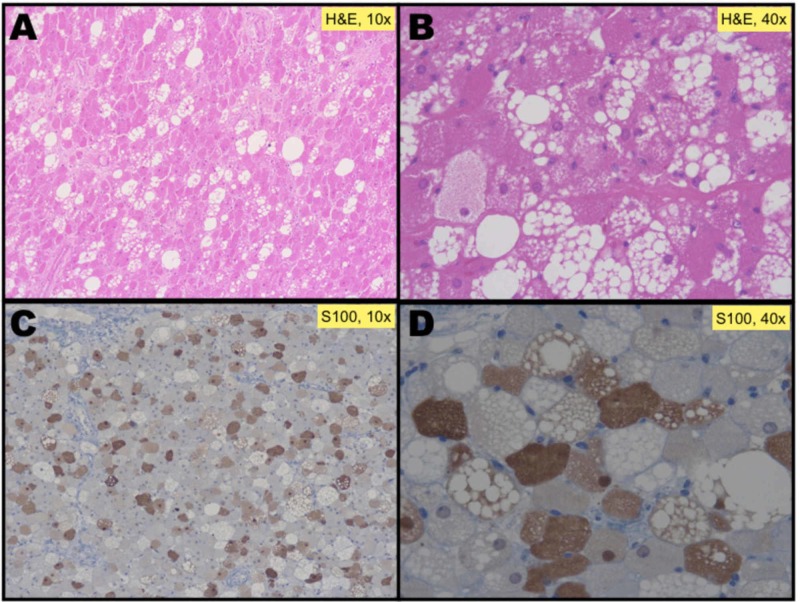
Histology from resected superior segment of the inferior lobe of the left lung. A. Under lower power 10x H&E. B. H&E 40x: round and polygonal cells with finely microvacuolated eosinophilic cytoplasm, and small central uniform nuclei, without visible mitosis, surrounded by scarce fibrovascular stroma, very similar to normal brown fat cells. C. Immunohistochemical staining protein S100 low power 10x. D. Immunohistochemistry protein S100 40x mainly highlighted the microvesicular brown cells. H&E, hematoxylin-eosin

On gross examination, the specimen was a lobulated mass with a smooth, tan to a brown external surface, 11 cm x 8 cm x 6 cm (longitudinal-anteroposterior-transverse) and weighed 195 g. Histological sections were cut and stained with hematoxylin-eosin (Figure 3A,B). Microscopic analysis revealed a lesion composed of round and polygonal cells with finely microvacuolated eosinophilic cytoplasm and small central uniform nuclei, without visible mitosis, surrounded by scarce fibrovascular stroma, very similar to normal brown fat cells. There is no presence of lipoblasts. Immunohistochemistry shows lesional cells expressing S100 protein, indicative of fatty infiltration (Figure 3C,D)..

## Discussion

A hibernoma is an uncommon benign soft tissue tumor composed of brown adipose cells.

These tumors represent 1.1% of all adipocytic tumors and at least 1.6% of the benign lipomatous tumors. Four morphologic variants have been identified: typical, myxoid, spindle cell, and lipoma-like [8]. Immunohistochemically, according to the World Health Organization, hibernoma cells are S100 positive in all variants and CD34 negative mostly [8-9]. Characteristic cytogenetic abnormalities described in hibernoma include structural rearrangements of 11q13 and 11q21 [10]. A slight male predominance has been reported as well as a higher incidence in adults with a mean age of occurrence of 38 years [8]. Hibernomas occur in a wide variety of locations, following the distribution of sites of the persistence of brown fat in adults. The majority of hibernomas are in the lower extremities, upper extremities, back, neck, mediastinum, peri-renal, and suprarenal sites. Intrathoracic hibernomas are very rare, accounting for only 0.065% of the cases in the most extensive review of hibernomas [7]. Clinically, these tumors are a slowly growing mass, firm, freely mobile, painless, and may rapidly increase in size. The tumors typically do not produce symptoms, unless they cause a mass effect upon adjacent structures [11-12].

Despite the use of multimodal imaging, the diagnosis of hibernoma is challenging. The diagnosis must be made based on the clinical, radiographic, and cytologic features.

In the first instance, this is an isolated posterior mediastinal mass. On CXR, the differential diagnosis is broad. This mass could be a paraspinal mass caused by a peripheral nerve sheath tumor such as schwannoma, neurofibroma, paraspinal ganglioneuroma. Other differential diagnoses of paravertebral masses are the paraspinal abscess, metastases, hematoma, descending aortic aneurysm, focal mass or dilatation of esophagus, duplication cyst, and extra-medullar hematopoiesis.

It is challenging to diagnose hibernoma through images alone. However, the radiologic features provide helpful information for differential diagnosis. Radiographs may show a consistent radiolucent mass, typically without areas of calcification or bone erosion [13]. The sonographic appearance shows a uniformly hyperechoic mass with hypervascularity and enlarged vessels [14]. The hypervascular nature of the tumor may lead to an erroneous diagnosis of malignancy. The CT images showed a heterogeneous mass with lower density areas (14 HU) indicating the presence of macroscopic fat; this could be the first clue to guide the diagnosis. Pre-contrast CT findings include well-circumscribed, heterogeneous lesions with mixed low-attenuation fatty components along with septae. Typically, enhancement of the septa and more diffuse uptake is characteristic after intravenous contrast injection, indicating internal vasculature [5-16]. The MRI features are not specific, high T1 signal intensity, although intermediate signal intensity has also been reported (intermediated between hyperintense compared to skeletal muscle and hypointense compared to subcutaneous fat) [14, 16]. The imaging characteristics of T2-weighted images demonstrated a lesion, which is nearly isointense with subcutaneous fat. On fat suppression sequences, there may be incomplete fat suppression because of the nature and amount of lipids [17].

Differential diagnosis of a mass with a signal similar to fat or containing large intratumoral vessels on CT or MRI should include benign and malignant lesions such as lipoma, angiolipoma, hemangioma, liposarcoma, well-differentiated liposarcoma, or atypical lipomatous tumors [13, 16]. 

A reliable diagnosis is difficult because hibernomas have a variable histologic appearance depending on the relative amount of multivacuolated, mitochondria-rich, and brown fat cells, which can alter the MRI signal characteristics [15]. Recently [18F] 2-deoxy-2-fluoro-D-glucose (FDG) positron emission tomography (PET) has become a useful tool to evaluate the functional characteristics of soft tissue masses. FDG-PET allows adipose tissue with a high rate of uptake of (18F-FDG) to be identified as brown adipose tissue, so it can be used to differentiate a hibernoma from a liposarcoma [18]. In this case, hibernoma shows higher standardized uptake value (SUV) (>10) than liposarcomas despite their benign nature. This can be attributed to the metabolically active cellular elements (more mitochondria with a higher rate of glucose metabolism) rather than reflect their malignant or not potential [18-19].

The histopathology is necessary for a diagnostic certainty; in these cases, a core needle biopsy can be performed with few complications despite their vascularity.

The treatment in most cases is complete surgical excision, which is generally curative and has a low potential of local recurrence; there is no conclusive evidence of a malignant form or metastatic disease, and their existence is debated with most reports dating back to the 1960s and 1970s. In the case reported by Lowry and Halmos, they argued malignancy by the mere fact of infiltration of the striated muscle by a typical hibernoma, which is inappropriate. While the three cases reported by Apatenko et al. histopathology reminds more a hibernoma but with larger and pleomorphic nuclei besides containing "strips reminiscent of liposarcoma or polymorphocellular sarcoma" and "immature fibroblastic tumor." The case reported by Teplitz et al. looked more like an anaplastic sarcoma (fascicular pattern of spindle cells with bizarre pleomorphic cells and atypical mitotic figures) with brown adipose tissue traces. Other authors such as Enterline et al. postulate the term of highly atypical hibernoma. Nevertheless, this case in their analysis does not rule out that it is a poorly differentiated liposarcoma by the widespread nuclear atypia in brownish areas and the low nuclear to cytoplasmic ratio [1, 7-8,19-20].

This patient after the tumor removal had a satisfactory postoperative evolution; the chronic cough resolved, and the patient had a steady follow-up course.

## Conclusions

Mediastinal hibernoma is a rare benign soft tissue tumor. The majority of cases occur in extremities with few cases with mediastinal localization. It should be suspected in cases of intra-thoracic fat density lesions in CT or MRI. The PET-CT can differentiate a hibernoma from a liposarcoma in case of doubts. The histopathology is necessary for a diagnostic certainty. It can be observed expectantly, or it can be excised when it becomes symptomatic, this being the definitive treatment with a low risk of recurrence.
